# Avoiding variability in sleep variability assessments: how many nights are enough?

**DOI:** 10.1093/sleep/zsag083

**Published:** 2026-03-26

**Authors:** Chun Siong Soon, Michael W L Chee

**Affiliations:** Centre for Sleep and Cognition, Yong Loo Lin School of Medicine, National University of Singapore (NUS), Singapore; Oura-National University of Singapore (NUS) Joint Lab, Oura Pte Ltd, Singapore; Centre for Sleep and Cognition, Yong Loo Lin School of Medicine, National University of Singapore (NUS), Singapore; Oura-National University of Singapore (NUS) Joint Lab, Oura Pte Ltd, Singapore

**Keywords:** sleep variability, sleep regularity, intraindividual variability, sleep trackers, wearables, sleep duration, sleep timing

Sleep variability—the night-to-night fluctuations in duration, timing, and efficiency of sleep—is emerging as an independent predictor of health outcomes, from cardiovascular disease to cognitive decline [1–4]. The rise of wearable sleep trackers increasingly empowers researchers to monitor sleep patterns in situ, over extended periods, and at scale. With such large-scale longitudinal objective data, we can now address a fundamental question that has lurked in the background for years: are we measuring sleep variability accurately [5]?

The answer, according to a landmark study by Leota and colleagues [6] published in this issue, is often no. Their analysis of over 3.7 million person-nights from more than 10 000 individuals reveals a sobering reality: the standard 1-to-2-week monitoring periods commonly used in accelerometry studies provide grossly inadequate estimates of sleep variability. While researchers can reliably capture average sleep duration in as few as 3 to 7 nights, accurately measuring night-to-night variability requires 6 to 10 weeks of continuous data—and even longer for individuals who are less consistent in providing wearable data.

## The Reliability Gap

The implications of this finding extend beyond methodological minutiae. Systematic reviews have consistently shown that sleep variability—quantified by the standard deviation (SD) or root mean square of successive differences across nights—independently predicts health outcomes better than what can be inferred from average sleep duration or timing [1, 7, 8]. Variable sleep has been linked to increased cardiovascular risk, metabolic dysfunction, mood disorders, and cognitive impairment. Yet if our measurements of this variability are unreliable, we may be systematically underestimating/attenuating the magnitude of true association through regression dilution.

Leota and colleagues addressed this reliability problem using massive amounts of data from WHOOP wearable devices worn continuously for 1 year. From this, they calculated reference values for sleep variability based on 363 nights of data per participant. They then systematically examined how shorter monitoring periods matched these reference values by randomly sampling 7 or 14 consecutive days—common actigraphy study durations. The results were striking: with 7 days of data, sleep variability measures (total sleep time, 3 timing metrics and wake after sleep onset) showed correlations of only 0.50–0.58 with their corresponding reference values. Doubling the duration to 2 weeks improved the correlations to 0.61–0.67— still well below the acceptable reliability threshold of 0.80.

Bland-Altman analyses painted an even less flattering picture of common current practices for assessing sleep variability. For total sleep time variability, a seven-night estimate could differ from the true value by up to 50 min in either direction. For sleep onset variability, the limits of agreement spanned over 2 h. These are not trivial measurement errors—they represent fundamental uncertainty about an individual’s sleep regularity profile.

## Implications for Major Sleep Studies

These findings cast a shadow over numerous influential datasets that have shaped our understanding of sleep and health. Consider the Multi-Ethnic Study of Atherosclerosis Sleep Study, which collected actigraphy data for 7 consecutive days from over 2000 participants [9]. This landmark study has generated dozens of publications linking sleep characteristics to cardiovascular outcomes. While the study’s measurements of average sleep duration are likely reliable based on the findings of Leota et al., any conclusions about sleep variability, including associations between irregular sleep and hypertension or cardiovascular events, may be substantially attenuated by measurement error.

Similarly, the Adolescent Brain Cognitive Development Study, one of the largest longitudinal studies of brain development and child health, has over 11 000 children enrolled. The study offers unprecedented opportunities to understand how sleep patterns influence neurodevelopment. However, actigraphy assessments of sleep were also collected over limited time windows [10].

Beyond these specific studies, a systematic review of sleep variability and health outcomes found dozens of studies that mostly rely on 1 to 2 weeks of actigraphy data [7]. If reliability coefficients for sleep variability hover around 0.60 for typical study durations, the observed correlations between sleep variability and health outcomes would be attenuated by approximately 40% compared to their true values. This means that the actual health impact of irregular sleep may be substantially larger than current literature suggests.

## Not All Sleep Metrics Are Created Equal

Importantly, Leota and colleagues reveal that reliability requirements vary dramatically across sleep parameters. Average sleep duration, timing, and fragmentation can be reliably estimated within three to seven nights—affirming the decades-long practice of 1–2 week data collection for these metrics [11–13]. The problem is specific to variability measures: SDs and successive differences that capture night-to-night fluctuations [5, 12].

The study also uncovered intriguing demographic differences. Women and younger adults required up to 30 additional days to achieve reliable estimates of sleep fragmentation variability compared to men and older adults respectively. Most strikingly, these gender differences disappeared after age 55, suggesting that menstrual cycle-related fluctuations may drive the additional variability in younger women [14]. These findings have profound implications for study design: a one-size-fits-all sleep variability assessment may systematically misrepresent certain demographic groups, introducing bias into our understanding of sleep health across populations.

## A Path Forward

What should researchers do with this information? The most straightforward action would be to extend monitoring periods when sleep variability is the sleep measure of interest. In addition to the specific guidance of 41–65 nights for reliable variability estimates across common sleep metrics, the authors have made publicly available an app (https://josh-leota.shinyapps.io/iiv-sleep-2025/) that helps researchers estimate required monitoring durations based on their specific metrics and reliability targets.

These recommendations notwithstanding, there are practical challenges to consider [12]. Longer monitoring periods increase participant impact, reduce adherence, and escalate costs. The authors’ replication analysis in a lower-compliance sample showed that irregular device wear itself necessitates even longer monitoring periods. Research teams must balance scientific rigor against feasibility.

For studies already completed with shorter monitoring periods, researchers should acknowledge the reliability limitations of their variability estimates and consider these when interpreting effect sizes [5]. There are also some workarounds: advanced statistical approaches such as location-scale models [15] or dynamic structural equation models [16] can partially account for measurement error in variability estimates. Sensitivity analyses comparing results across different monitoring durations could facilitate the discernment of the more robust findings.

For new studies involving sleep variability, researchers should carefully consider the new recommendations, with particular attention adjusting these according to demographic characteristics. Studies focusing solely on sample averages in sleep measures can continue to use shorter protocols, but studies focused on understanding inter-individual differences must collect data for the longer, recommended durations.

## Looking Ahead

This study exemplifies how wearable technology is simultaneously creating new research opportunities and exposing limitations in existing data collection practices. The message is clear: when it comes to measuring sleep variability in naturalistic free-living conditions, 1 or 2 weeks of data is insufficient. As sleep regularity gains increasing recognition as a vital indicator of sleep health, our methods must evolve accordingly. The question “how many nights are enough?” now has an answer that is empirically grounded, and it’s considerably more than most of us have been collecting.

## Disclosure statement

*Financial disclosure*: The authors are part of the Oura-NUS joint lab, but the contents of this commentary are independent of any commercial interest.

*Non-financial disclosure:* Michael W. L. Chee is a member of the Medical Advisory Board of Oura Health. The statements and opinions expressed are solely the responsibility of the author and do not represent the official views of Oura Health Oy.
